# Is prophylactic administration of the anticonvulsants necessary in tramadol-intoxicated patients after an initial seizure?

**DOI:** 10.1186/2008-2231-21-60

**Published:** 2013-07-24

**Authors:** Hossein Sanaei-Zadeh

**Affiliations:** 1 Emergency Room/Division of Medical Toxicology, Hazrat Ali-Asghar (p) Hospital, Shiraz University of Medical Sciences, Shiraz, Iran

## Letter to the editor

Tramadol is nowadays one of the most commonly abused drugs in Iran. In our country, few emergency physicians have never visited a tramadol-intoxicated patient referring after overdose or with side effects of its use. One of the complications of tramadol use, abuse, or overdose is seizure [1-4]. It has been shown that the tramadol-induced seizures are not dose-dependent [5]. To date, the incidence rate of recurrent/multiple seizures in tramadol-intoxicated patients has been determined in several studies as shown in Table 1[3,6-11]. The important question is that is there a need for prophylactic administration of anticonvulsants in tramadol-intoxicated patients referring with an initial seizure? Of note, in some poison treatment centers of Iran, administration of benzodiazepine is a routine treatment in such patients. As you know, in the setting of clinical toxicology, except for the intravenous overdose of vincristine [12], severe toxicity with methylxanthines [13], strychnine toxicity [14], and withdrawal of ethanol and some benzodiazepines [15], no other condition exists for whose prevention of recurrent or multiple seizures, prophylactic anticonvulsants have been recommended [16]. This certainly applies to the tramadol-induced seizures, as well, because these seizures have no special characteristic for which we believe them to be different from other drug- and toxin-induced seizures. For instance, tramadol-induced seizures are tonic-clonic, short-lived, and self-limited similar to the most of other drug-induced seizures [16]. In addition, in the setting of poisoning, even where there is the likelihood of recurrent seizures (except for the above-mentioned conditions), prophylactic administration of the anticonvulsants has not been recommended and in the case of development of such seizures, they are treated similarly to a single episode [16]. Therefore, prophylactic administration of anticonvulsant appears to be unnecessary even if the patients have an initial seizure. In addition, the cost-effectiveness of such unnecessary treatment should not be forgotten. Also, as you know, there is not a standardized management protocol for tramadol toxicity. For example, with respect to the possibility of the occurrence of seizure or apnea, how long does the physician need to observe the patients? Should activated charcoal be administrated after tramadol overdose? [17] Should naloxone be given to the comatose tramadol-intoxicated patients? [2,3,18,19] This highlights the role of global act by doing more research to fix the problem of tramadol [20,21].

**Table 1:** Sample of the published articles related to the incidence of recurrent/multiple seizures

**Authors**	**Publication year**	**Number of the patients studied**	**Incidence of the recurrent/multiple seizures**
Spiller et al. [3]	1997	87	1.1%
Jovanović-Cupić et al. [6]	2006	57	55%
Petramfar et al. [7]	2010	106	1.9%
Taghaddosinejad et al. [8]	2011	401	24%
Gudarzi et al. [9]	2011	54	35%
Farajidana et al. [10]	2012	232	10.8%
Shadnia et al. [11]	2012	100	7%
